# Dental Pathology

**Published:** 1883-08

**Authors:** J. M. Clide


### Dental Pathology.

BY DR. J. M. CLIDE.

[Read before the Kentucky State Dental Society.]

This, probably the most difficult and least understood of all the medical sciences, has been assigned to me and my friend, Dr. Cooper, and that, too, without limitation. It is difficult to separate pathology and etiology. Indeed, Greene, in his introduction, says pathology treats of the origin, nature, course and cause of those changes in the body which constitute disease. This is, I think, a very broad and comprehensive definition, and yet it is not easy, or perhaps judicious, to restrict it. Physiological and pathological conditions are somewhat antipodal. A tissue or organ is in a physiological condition when it performs its functions perfectly. This is its normal condition. Whenever an organ fails in any degree to perform its functions in harmony, it is just to that extent in an abnormal or pathological state. This, were the definition restricted within its narrowest limits, is the thing to be studied under the head of pathology. But this would leave us in ignorance as to the proper therapeutical agents, and hence a knowledge of the origin and causes of the disease in question is necessary in order to an intelligent application of these remedies.

Having made these general remarks, let us direct attention for a short time to a few special cases. First, dental caries. Here the origin and cause, whether acids or bacteria, are still matters of dispute. We believe the acid theory accounts better than the other for the different conditions, whether white, light, brown, or black, soft, leathery, or hard. But, whatever may be the, cause we know that the remedy consists in a thorough use of the excavator, and the substitution of gold or some other material, and in the application of such constitutional and local remedies as will neutralize the acid conditions or destroy the bacteria, which produce decay.

Next, alveolar ulceration. I believe it is generally held that this begins at the margin of the gums and is caused by calcareous deposits about the neck of the teeth. This may be true, but may not the real cause lie in a too low grade of dental vitality, a grade of life so far inferior to that of the surrounding tissues, that nature, ever true to herself, failing to restore the lost vitality, sets this process in operation to divest herself of those organs which have become to all intents and purposes, irritants? I ask this question merely to provoke discussion and not from any real investigation I have made, or conviction that such is the true cause. That the deposit on the roots of such teeth differs in some respects from that about the necks is patent to the observation of every dentist. It adheres very firmly to the roots and is granular, that is, it is not deposited evenly on the roots like salivary calculus about the necks, but in nodules scattered more or less thickly, so far as the disease extends. It is also accompanied by an almost constant discharge of a greater or less amount of degenerated pus, very offensive, especially to the operator. Salivary calculus, I believe, generally destroys the entire gum so far as the deposit extends, but alveolar ulceration more especially destroys the bony tissue. I have seen the soft parts left almost in sight, the bony walls which support the teeth almost entirely gone. As a natural consequence, the gums in such a condition are more or less congested, flabby, and tumefied. I have seen all this where there was but little deposit of salivary calculus. Now I wish to ask is alveolar ulceration the result of the deposit, or is the deposit a result of the ulceration and chemical analysis of the pus found? Does this pathological state have its beginning, progress, and end in salivary calculus, or is it a disease *per se?* Can this disease begin around the root of a tooth which is not in itself in a pathological condition? And, lastly, is not this the condition known by the high-sounding and euphonious title, *pyorrhœa alveolaris?*

---

A MAN in England passed fæces swarming with maggots. The "Britishers" were for a time much puzzled, but concluded finally that the butter which the man ate contained maggots. If this startling case had occured on this side of the Atlantic we should have said the patient had taken a glass of soda with a "fly" in it.

